# Effect of a resistance exercise at acute moderate altitude on muscle health biomarkers

**DOI:** 10.1007/s00424-023-02868-y

**Published:** 2023-10-11

**Authors:** Sergio Pérez-Regalado, Josefa León, Paulino Padial, Cristina Benavente, Jose D. Puentes-Pardo, Filipa Almeida, Belén Feriche

**Affiliations:** 1 Department of Physical Education and Sport, Faculty of Sport Sciences, University of Granada, 18011 Granada, Spain; 2 Clinical Management Unit of Digestive System, San Cecilio Hospital, Ibs.GRANADA, 18016 Granada, Spain

**Keywords:** Hypoxia, Resistance training, Altitude, Inflammation, Cytokines

## Abstract

The intensification of the stress response during resistance training (R_T_) under hypoxia conditions could trigger unwanted effects that compromise muscle health and, therefore, the ability of the muscle to adapt to longer training periods. We examined the effect of acute moderate terrestrial hypoxia on metabolic, inflammation, antioxidant capacity and muscle atrophy biomarkers after a single R_T_ session in a young male population. Twenty healthy volunteers allocated to the normoxia (N < 700 m asl) or moderate altitude (HH = 2320 m asl) group participated in this study. Before and throughout the 30 min following the R_T_ session (3 × 10 reps, 90 s rest, 70% 1RM), venous blood samples were taken and analysed for circulating calcium, inorganic phosphate, cytokines (IL-6, IL-10 and TNF-α), total antioxidant capacity (TAC) and myostatin. Main results displayed a marked metabolic stress response after the R_T_ in both conditions. A large to very large proportional increase in the adjusted to pre-exercise change of inflammatory and anti-inflammatory markers favoured HH (serum TNF-α [ES = 1.10; *p* = 0.024] and IL-10 [ES = 1.31; *p* = 0.009]). The exercise produced a similar moderate increment of myostatin in both groups, followed by a moderate non-significant reduction in HH throughout the recovery (ES =  − 0.72; *p* = 0.21). The R_T_ slightly increased the antioxidant response regardless of the environmental condition. These results revealed no clear impact of R_T_ under acute hypoxia on the metabolic, TAC and muscle atrophy biomarkers. However, a coordinated pro/anti-inflammatory response balances the potentiated effect of R_T_ on systemic inflammation.

## Introduction

**Fig. 1:**
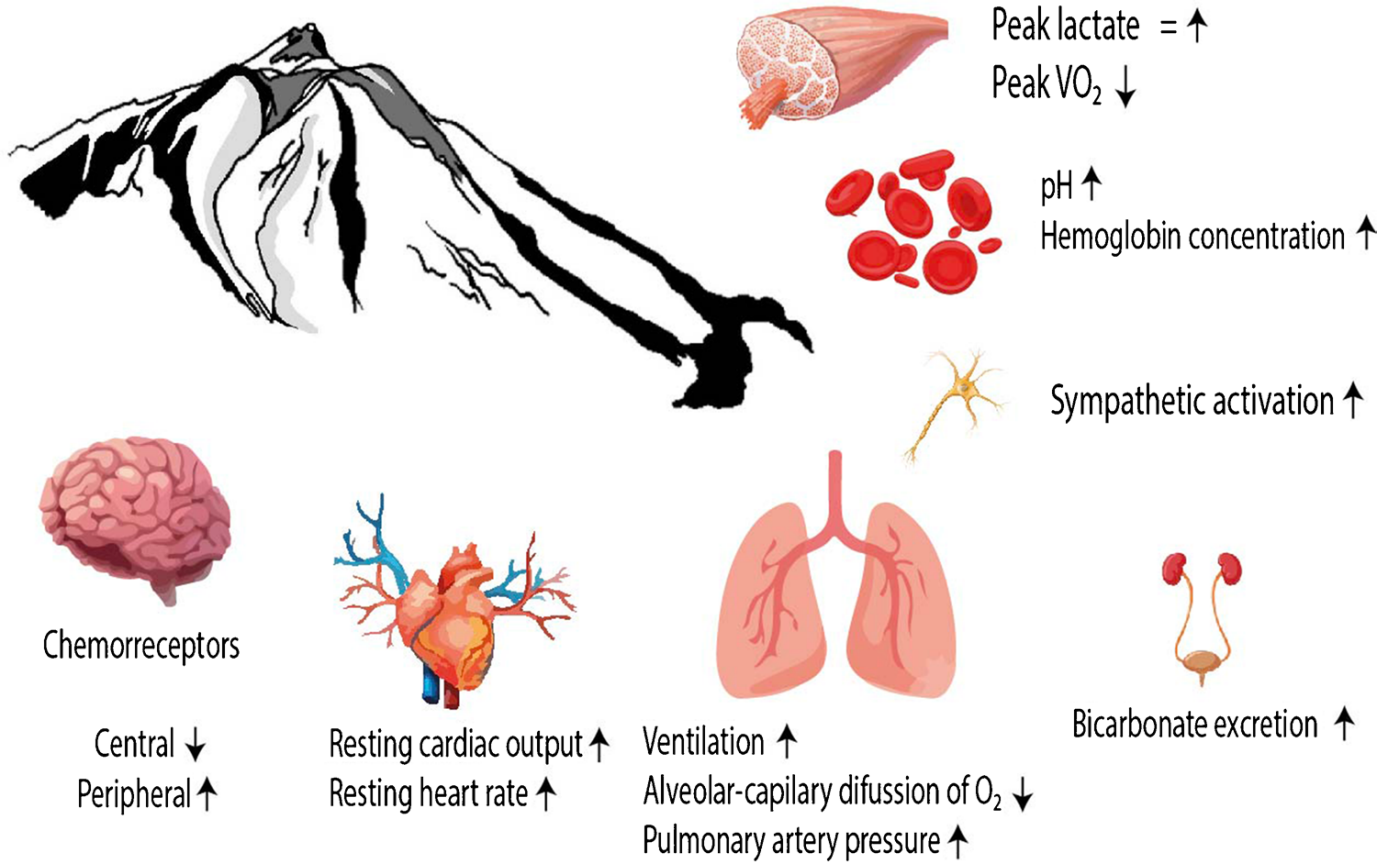
Schematic representation of acute physiological effects of altitude exposure

In recent decades, the scientific community has focused on understanding the benefits of hypoxia exposure on sports performance. At moderate terrestrial hypobaric hypoxia (HH), athletes can benefit from the stimulus of hypoxia, avoiding the adverse effects that could accompany the ascent and staying at higher altitudes [3]. The most sought-after adaptations are haematological, primarily related to long-distance disciplines due to their potential linkage to haemodynamic adaptation, involving vasodilation and blood flow improvement [27] (Fig. 1). Conversely, research on resistance training (R_T_) under hypoxic conditions on muscle mass development is inconclusive and quite scarce when it comes to the overall well-being and functionality of skeletal muscle (muscle health) analysis [8].

A cascade of inflammatory events initiates during and after acute resistance exercise in hypoxia [18, 38]. The inflammation constitutes a significant source of reactive oxygen and nitrogen species (RONS) and plays an essential role in skeletal muscle repair after exercise-induced muscle injury [25]. Furthermore, RONS derived from R_T_ may contribute to enhance muscle protein synthesis and reduce muscle damage via the modulation of antioxidant signalling pathways [15]. Tissue hypoxia and RONS are involved in the activation of hypoxia-inducible factor-1α (HIF-1α), which has a key function in immune cell mobilisation and activation of cell survival mechanisms in skeletal muscle [21, 24]. Furthermore, HIF-1α stabilisation leads to inflammation and directly modulates the secretion of pro-inflammatory cytokines, such as tumour necrosis factor-alpha (TNF-α) and the interleukin-6 (IL-6)/soluble IL-6 receptor (sIL-6R) complex, among others [23].

TNF-α is synthesised by multiple immune cells and rapidly released into the blood after acute R_T_ [29, 37]. Several authors have suggested that TNF-α activation is an early mediator of muscle damage and apoptosis, protein degradation and promotion of the inhibition of satellite cell differentiation via upregulation of the inflammatory response and myostatin expression [35]. Myostatin is a member of the transforming growth factor-beta superfamily secreted in skeletal muscle, among other tissues, due to oxidative stress, inflammation and glucocorticoid stimuli, and acts as a negative regulator of skeletal muscle mass [40]. It has also been shown that overexpression of inflammation-related proteins increases intracellular RONS and may induce cellular and extracellular damage due to a feedback loop in the TNF-α/myostatin axis [35] being expected that hypoxia further stimulates inflammation compared with normoxia [20]. It has also been demonstrated that chronic inflammatory status is associated with several diseases, including sarcopenia, which leads to progressive loss of muscle mass, highlighting the crucial role of the pro/anti-inflammatory balance in muscle health status [13].

Muscle contractions from acute R_T_ increase and mobilise inflammatory cytokines, such as IL-6 [30] and immune cells, followed by the release of anti-inflammatory markers (IL-10, IL-1ra and sTNF-R1) [12]. Although IL-6 is considered an inflammatory cytokine, there is an association between satellite cell proliferation and transitory augments of IL-6, while chronic elevation is negatively associated with muscle health [8]. Furthermore, it also participates in the transition to an anti-inflammatory phenotype [4, 28] by inducing the subsequent release of IL-10, a key factor in the upregulation and control of the anti-inflammatory response [39]. IL-10 leads to positive self-feedback via ROS-dependent responses [22], preventing an exacerbation of the inflammatory state. However, the mechanisms through which the antioxidant response modulates the inflammatory system during R_T_ performed under hypoxic conditions remain unknown. A recent meta-analysis on the topic concluded that exercise in hypoxia does not stimulate to a greater extent IL-6 or TNF-α than normoxia but leads to an increment in IL-10 [20]. However, this meta-analysis mixed terrestrial and normobaric hypoxia (NH) studies of different severity and duration with different types of exercise (i.e. the majority of the 23 included studies used aerobic exercise, and only 3 of them referred to R_T_ exercise). Interestingly, the physiological impact of exercise in terrestrial hypoxia is described as more severe than NH for the same effective FiO_2_ [31]. Moreover, data from R_T_ under acute exposition to terrestrial hypoxia constitute scarce but very useful information on muscle stress response during the acclimatisation phase, critical training days during altitude training camps.

Therefore, this research aimed to compare the acute effect of a single R_T_ session on exercise-induced metabolic stress (such as circulating calcium and inorganic phosphate), inflammation (IL-6 and TNF-α), total antioxidant capacity and muscle mass atrophy (myostatin) biomarkers in normoxia and moderate HH. We hypothesised that HH will favour higher accumulation of stress-derived metabolites after R_T_ exercise, thus impacting total antioxidant capacity (TAC) and inflammatory response than the same exercise in N.

## Materials and methods

### Design

A cross-sectional design with two independent controlled groups and intra-/inter-group measurements was used to analyse the acute effect of a traditional hypertrophy training session [9] on metabolic stress, inflammation, antioxidant capacity and muscle atrophy biomarkers. Following the successful completion of baseline assessments, the participants were aleatory allocated to the N or HH group (HH 2320 m asl; N 690 m asl) (Table 1).

**Table 1:** Descriptive characteristics of study participants

Parameter	HH (*n* = 10)	N (*n* = 10)
	Mean ± SD	Mean ± SD
Age (years)	22.8 ± 4.24	22.7 ± 3.37
Body mass (kg)	74.03 ± 13.87	72 ± 7.70
Height (cm)	177.3 ± 7.40	175.3 ± 4.11
Fat mass (%)	9.38 ± 4.81	10.68 ± 3.98
BMI (kg/m^2^)	23.46 ± 4.14	23.42 ± 2.30

*N* normoxia, *HH* hypobaric hypoxia, *SD* standard deviation, *BMI* body mass index

### Participants

Twenty physically active Caucasian male volunteers participated in this study. All participants had performed R_T_ for a minimum of three times per week for at least the previous year. The participants had no health or muscular disorders, reported that they had not consumed any agents associated with muscle size development during the previous month and were unacclimated to high altitude (no altitude exposure in the 2 months before the study). All participants lived in N and were exclusively exposed to altitude during the training session. This study was approved by the local Ethics Committee (PEIBA: 2212-N-21) and was conducted in accordance with the Declaration of Helsinki and Biomedical Research (14/2007). Informed written consent was obtained from all participants prior to beginning the study.

### Procedure

One week before the R_T_ session, the participants engaged in a preparatory session to determine the training load (70% of 1 repetition maximum [1RM]) for each exercise. After a specific warm-up, the participants performed 1 set at the perceived 75% of 1RM (10 RM) followed by 1–2 additional sets with increasing loads until performing 2–3 repetitions to failure. The rest period between sets was kept to 5 min. 1RM values were predicted using Brzycki’s equation [6]. The participants rested for 72 h between the training session and refrained from performing any additional resistance-type or high-intensity training throughout the duration of the study. Two days before the beginning of the study, the participants visited the laboratory for baseline anthropometric measurements (height [Seca 202, Seca Ltd., Hamburg, Germany] and body mass [Tanita TBF-300, Tokyo, Japan]) after they had fasted since midnight of the previous day. Testing sessions were conducted at the same time of day, at a temperature of ~ 22 °C and ~ 60% humidity for the N condition or ~ 22 °C and ~ 28% humidity for the HH condition. The participants travelled by car to the HH training centre (32 km) to perform the training session. Arrival at the HH training centre occurred ~ 30 min before the training began. Arterial oxygen saturation (SpO_2_; Onyx Vantage 9590; Nonin, Plymouth, MN, USA) was assessed per duplicate before the start of the warm-up of the R_T_ session to test the HH condition. Sensors displayed high reliability mean SpO_2_ values of 93.9% ± 1.63% and 97.2% ± 1.25% (CV < 1.74%), respectively, for the HH and N condition (*p* < 0.001).

The R_T_ session comprised 3 sets of 8–10 repetitions at 70% 1RM of six functional exercises involving the main muscle groups of the body (full-body routine: back squat, deadlift, seated cable row, wide grip lat pulldown, bench press and barbell military press). The participants rested 90 s between each set. A standardised warm-up of 15 min was completed at the beginning of the training session (10 min of low-intensity aerobic exercise and stretching exercises followed by a specific warm-up of 2 sets of 10 repetitions [the first with 20 kg and the second at 50% 1RM estimated from the preliminary test, 120 s rest] of the back squat, seated cable row and bench press).

### Blood samples

Blood samples were taken throughout the 30 min following the R_T_ session to analyse the kinetics of release/clearance of the studied variables in this time window. Serum circulating variables, such as metabolites, inorganic phosphate (Pi) and calcium (Ca_2+_), TAC and cytokines (IL-6, IL-10, TNF-α and myostatin), were determined. Immediately following the exercise, an antecubital vein of the arm was canalised via a catheter and remained permeable by using a physiological saline solution. Two millilitres of blood before each extraction were discarded to avoid dilution of the sample. Five millilitres of blood were extracted at 5, 10 and 30 min after the R_T_ session. All blood samples were kept in cold conditions after extraction and centrifuged to isolate serum within 4 h at 3000 rpm for 10 min. Finally, several 500 µl aliquots of serum were stored at − 70 °C until use. The basal condition was established before the beginning of the study at N conditions from a blood collection after two days of refraining from any exercise.

Cytokines were assessed at 5 and 30 min following the R_T_ session using the Milliplex Human High Sensitivity T Cell Panel (HSTCMAG-28SK) from Sigma-Aldrich (Darmstadt, Germany). The assay sensitivity for cytokines is 0.11–8.17 pg/ml. Myokine analyses were performed using the GDF-8/Myostatin Quantikine ELISA Kit (DGDF80) from R&D Systems (Minneapolis, MN, USA). The detection range of the Myostatin kit is 0.922–5.32 pg/ml. Antioxidant and metabolic response analyses (TAC, Pi and Ca^2+^) were assessed at 5, 10 and 30 min following the R_T_ session. TAC was determined using a Total Antioxidant Capacity Assay Kit (MAK187-1KT) from Sigma-Aldrich. The detection range of the kit is 4–20 nmol/µl. Quantitative data were obtained using the Luminex-200 system (Luminex Corporation, Austin, TX, USA), and data analysis was performed with Luminex 100™ IS v2.3 software. Serum Pi and Ca^2+^ were analysed in a COBAS C-311 instrument from Roche (Indianapolis, IN, USA). The detection range of the kits is 0.62–5.54 mmol/l for Pi and 0.28–4.65 mmol/l for Ca^2+^. All procedures followed the manufacturers’ instructions. All analyses were performed in normoxia using the same equipment by specialised staff.

### Statistical analyses

The data are generally presented as the mean ± standard deviation (SD), except the figures that present them as median and interquartile range. Shapiro–Wilk test was used to determine data distribution. The variables that did not follow a normal distribution were subjected to a transformation process. To determine the presence of outliers in the raw data, we employed the three-sigma rule of thumb method [2].

Firstly, differences between the environmental condition and time following the R_T_ session were interpreted through repeated measures analysis of variance (ANOVA). A two-factor ANOVA with repeated measures was used to assess the effect of time during the recovery (within-group factor with 3 levels [minutes 5, 10 and 30]) and the environmental condition (inter-group factor with 2 levels [HH vs. N]) on the ions (i.e. Pi and Ca_2+_) and TAC. A second two-factor ANOVA with repeated measures was used to assess the effect of time during the recovery (within-group factor with 2 levels [minutes 5 and 30]) and the environmental condition (inter-group factor with 2 levels [HH vs. N]) on the cytokines (i.e. IL-6, TNF-α, IL-10 and myostatin). The pre-test value was included as a covariate to control participant variability. Significant main effects and interactions were subsequently analysed with Bonferroni post hoc tests. When the sphericity assumption for ANOVA was violated, we applied the Greenhouse–Geisser correction. Partial eta squared for main effects was calculated from the ANOVA (*η*^2^_*p*_) and was interpreted as ≥ 0.01 (small), ≥ 0.06 (medium) and ≥ 0.14 (large) [7]. Significant main effects and interactions were subsequently analysed with the Bonferroni post hoc test.

Secondly, paired-sample *t* tests were used to evaluate the within-group exercise effect (pre-exercise vs. peak post-exercise value) for each variable. Complementary independent-sample *t* tests were used to identify differences in the exercise effect (absolute delta changes [peak post-exercise value–pre-exercise value]) between environmental conditions (HH vs. N) for each variable. The IL-10/TNF-α ratio was also compared between HH and N and interpreted as a systemic pro/anti-inflammatory index [34].

Complementary to the previous tests (repeated measures ANOVA and *t* test), the magnitude of the changes was quantified using the standardized differences based on Cohen’s effect sizes (ES). These measures were calculated as the mean change (HH-N; 30–10 min or 10–5 min or 30–5 min; and post–pre exercise values) divided by the pooled SD for all pairs comparisons. The thresholds for interpretation of Cohen’s *d* were set as follows: < 0.2 (trivial), 0.21–0.5 (small), 0.5–0.8 (moderate), 0.8–1.3 (large) and > 1.30 (very large) [17]. All analyses were performed using IBM SPSS Statistics version 28.0.1.0 for MacOS (IBM Corp., Armonk, NY, USA). The level of significance was set at *p* < 0.05.

## Results

The results from intra-group (extreme post-exercise value vs. pre-exercise) and inter-group (HH vs. N) exercise effects are presented in Table 2. Compared with pre-exercise, R_T_ session increased serum TAC levels in N (ES > 0.93 [95% CI 0.21, 1.62]; *p* = 0.011), and myostatin (ES > 0.75; all *p* < 0.041) and circulating Ca^2+^ in both conditions in a similar magnitude (ES > 1.16; all *p* < 0.005). Compared with N, HH showed a very large increase in IL-10 (ES = 1.31 [95% CI 0.32, 2.27]; *p* < 0.009). Similarly, exercise in HH maintained elevated TNF-α (ES = 1.10 [95% CI 0.14, 2.03]; *p* = 0.024) after R_T_ with respect to N, while IL6 displayed no changes throughout the 30 min of recovery. Moreover, there was a moderate but non-significant exercise effect between conditions for TAC and Pi. IL-10/TNF-α ratio showed large increments in both groups after exercise (ES > 1.36; all *p* < 0.002) with no differences between them (ES = 0.02 [− 0.86, 0.90]; *p* = 0.96).

**Table 2:** Exercise impact on inflammation and metabolic stress biomarkers

Variable	Env. condition	Pre ± SD	Peak value ± SD	*p*-value **peak value vs. pre**	ES [IC 95%]	Δ peak value ± SD	*p*-value **HH vs. N**	ES [IC 95%]
IL-6 (pg/ml)	N	45.68 ± 57.62	20.90 ± 27.25	*p* = 0.51	− 0.22 [− 0.84,0.41]	− 21.83 ± 26.52	*p* = 0.98	0.008 [− 0.88, 0.87]
	HH	50.77 ± 60.38	52.34 ± 74.77	*p* = 0.32	0.33 [− 0.96, 0.31]	2.22 ± 17.74		
IL-10 (pg/ml)	N	13.68 ± 6.19	11.14 ± 9.43	*p* = 0.12	− 0.50 [− 1.09, 0.12]	− 2.29 ± 8.16	***p* = 0.009**	**1.31** [0.32, 2.27]
	HH	24.90 ± 17.39	33.00 ± 19.01	***p* = 0.028**	**0.75** [0.08, 1.40]	8.10 ± 7.72		
TNF-α (pg/ml)	N	31.33 ± 11.37	21.71 ± 4.08	***p* = 0.021**	** − 0.89** [− 1.61, − 0.13]	− 9.61 ± 11.96	***p* = 0.024**	**1.10** [0.14, 2.03]
	HH	32.83 ± 10.00	52.33 ± 74.77	*p* = 0.21	0.43 [− 0.23, 1.07]	17.93 ± 33.04		
IL10/TNF-α ratio (pg/ml)	N	0.44 ± 0.17	0.49 ± 0.33	***p* < 0.001**	**2.43** [1.15, 3.68]	0.04 ± 0.20	*p* = 0.96	0.02 [− 0.86, 0.90]
	HH	0.80 ± 0.60	0.84 ± 0.71	***p* < 0.002**	**1.36** [0.46, 2.22]	0.05 ± 0.36		
Myostatin (pg/ml)	N	2033.47 ± 2283.35	5382.15 ± 5452.99	***p* = 0.021**	**0.88** [0.12, 1.60]	3384.62 ± 5773.10	*p* = 0.74	− 0.15 [− 1.02, 0.73]
	HH	2911.33 ± 2645.63	4270.73 ± 3568.86	***p* = 0.041**	**0.75** [0.03, 1.45]	1004.98 ± 1728.49		
Calcium (mmol/L)	N	2.34 ± 0.06	2.48 ± 0.10	***p* = 0.005**	**1.16** [0.33, 1.96]	0.14 ± 0.12	*p* = 0.53	− 0.29 [− 1.16, 0.60]
	HH	2.39 ± 0.06	2.50 ± 0.07	***p* < 0.001**	**2.01** [0.89, 3.09]	0.12 ± 0.07		
Inorganic phosphate (mmol/L)	N	1.39 ± 0.09	1.07 ± 0.25	***p* = 0.004**	** − 1.22** [− 2.03, − 0.37]	− 0.86 ± 0.16	*p* = 0.12	0.73 [− 0.19, 1.63]
	HH	1.18 ± 0.18	1.04 ± 0.19	*p* = 0.10	− 0.58 [− 1.24, 0.10]	− 0.68 ± 0.21		
TAC (pg/ml)	N	0.72 ± 0.03	0.77 ± 0.05	***p* = 0.011**	**0.93** [0.21, 1.62]	0.05 ± 0.05	*p* = 0.21	− 0.58 [− 1.46, 0.33]
	HH	0.77 ± 0.03	0.78 ± 0.07	*p* = 0.57	0.17 [− 0.40, 0.74]	0.01 ± 0.08		

Data presented as mean ± SD

*Δ peak value* absolute delta change (difference between the peak post-exercise value–pre-exercise value) between environmental conditions (HH vs. N), *Env. condition* environmental condition, *Peak value* extreme value throughout the recovery, *Pre* basal condition, *N* normoxia, *HH* hypobaric hypoxia, *ES* effect size, *CI* confidence interval, *SD* standard deviation. The data in bold are the values that show statistical significance

**Fig. 2:**
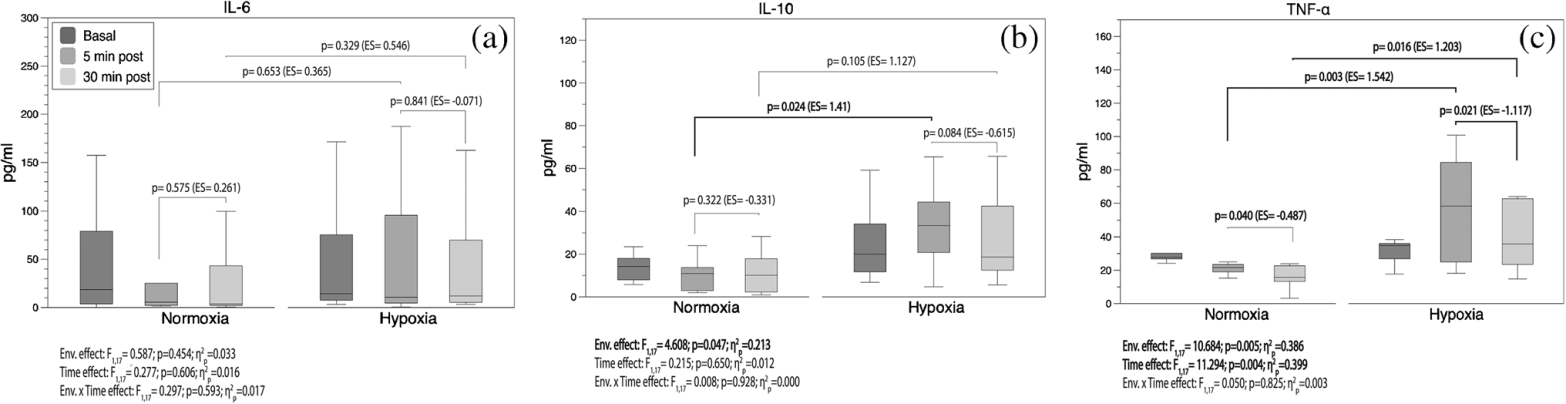
Analysis of circulating cytokines IL-6 (**a**), IL-10 (**b**), and TNF-α (**c**) response throughout the 30 min of recovery. Boxes represent the median and interquartile range, whiskers minimum to maximum values. *p*-value (*p* < 0.05), partial eta squared from ANOVA (*η*^2^_*p*_), Cohen’s *d* effect size (ES). ES was calculated as (30–5 min post-exercise) or (HH-N) divided by the pooled standard deviation. Pre-exercise values were included as covariates

The serum cytokine recovery kinetics results are shown in Fig. 2a–c. IL-6 displayed large individual variability, which made it difficult to detect any change (Fig. 2a). Contrary, TNF-α displayed an environmental (*F*_1,17_ = 10.684; *p* = 0.005; *η*^2^_*p*_ = 0.39) and a time effect (*F*_1,17_ = 11.294; *p* = 0.004; *η*^2^_*p*_ = 0.40) during recovery (Fig. 2c). Pairwise comparisons revealed large increases for TNF-α in HH at 5 and 30 min following the R_T_ session (all *p* < 0.05) (Fig. 2c). IL-10 only displayed an environmental effect during recovery (*F*_1,18_ = 4.608; *p* = 0.047; *η*^2^_*p*_ = 0.21). The pairwise comparison test yielded a large increase in IL-10 favouring HH at 5 min after the R_T_ (*p* = 0.02) (Fig. 2b).

**Fig. 3:**
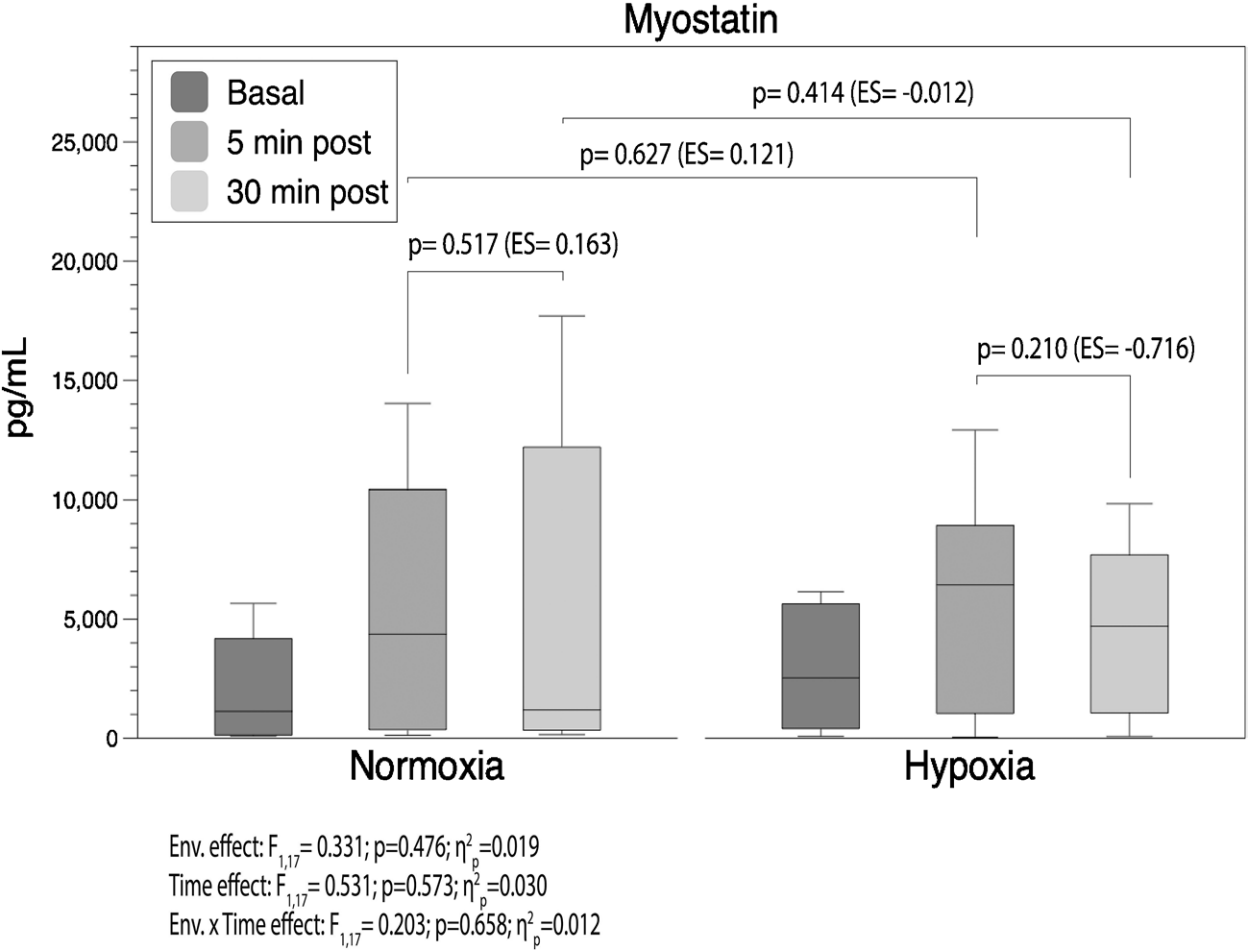
Analysis of circulating myostatin throughout the 30 min of recovery. Boxes represent the median and interquartile range, whiskers minimum to maximum values. *p*-value (*p* < 0.05), partial eta squared from ANOVA (*η*^2^_*p*_), Cohen’s *d* effect size (ES). ES was calculated as (30–5 min post-exercise) or (HH-N) divided by the pooled standard deviation. Pre-exercise values were included as covariates

Figure 3 displays the box plots of serum myostatin levels. Post-exercise results revealed a moderate but nonsignificant reduction in HH conditions at minute 30 of the recovery (ES =  − 0.71; *p* = 0.21).

**Fig. 4:**
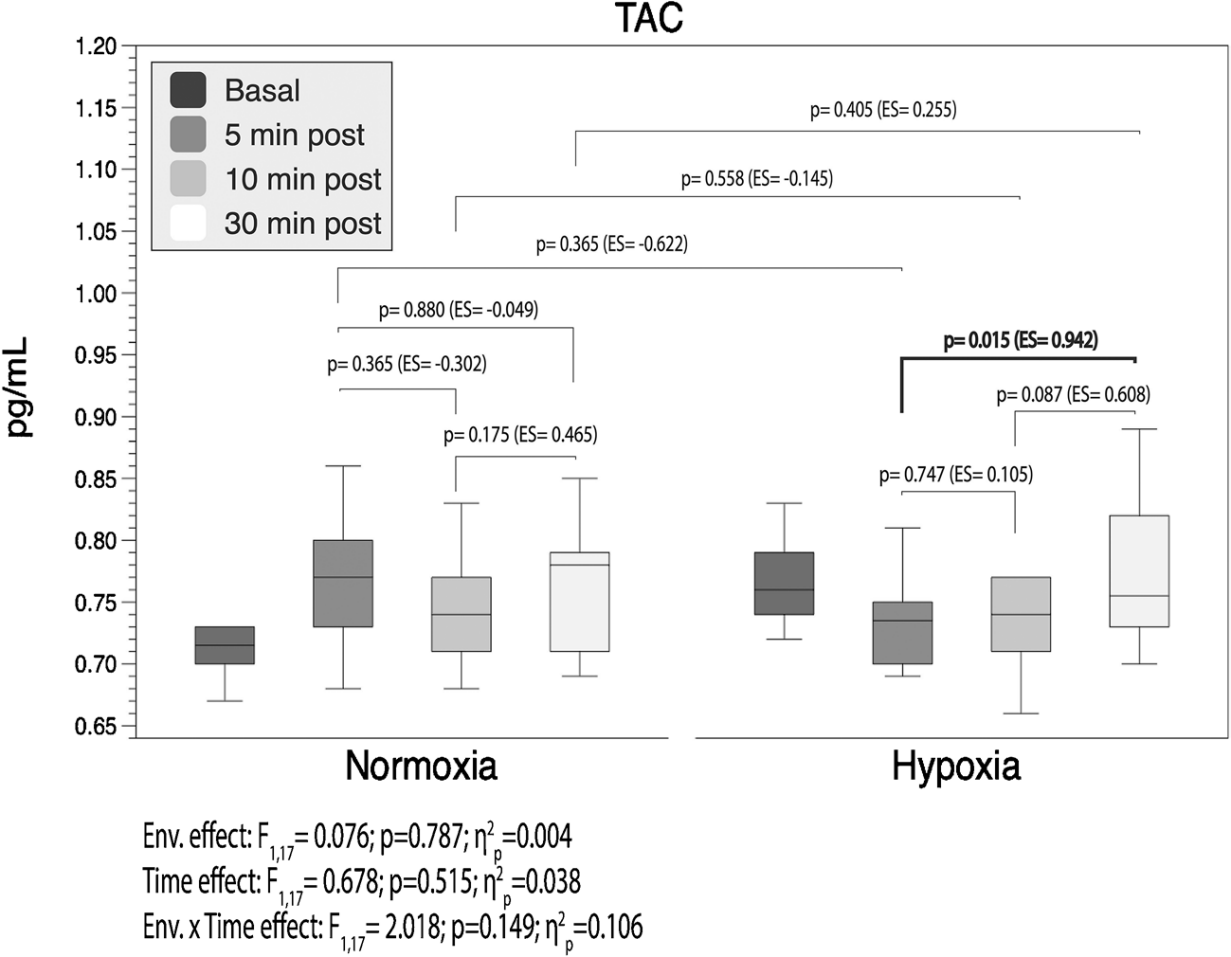
Total antioxidant capacity changes throughout the 30 min of recovery. Boxes represent the median and interquartile range, whiskers minimum to maximum values. *p*-value (*p* < 0.05), partial eta squared from ANOVA (*η*^2^_*p*_), Cohen’s *d* effect size (ES). ES was calculated as (30–10 or 10 − 5 or 30 − 5 min post-exercise) or (HH-N) divided by the pooled standard deviation. Pre-exercise values were included as covariates

TAC serum levels are shown in Fig. 4. Results displayed similar post-exercise results between conditions (*p* > 0.05).

**Fig. 5:**
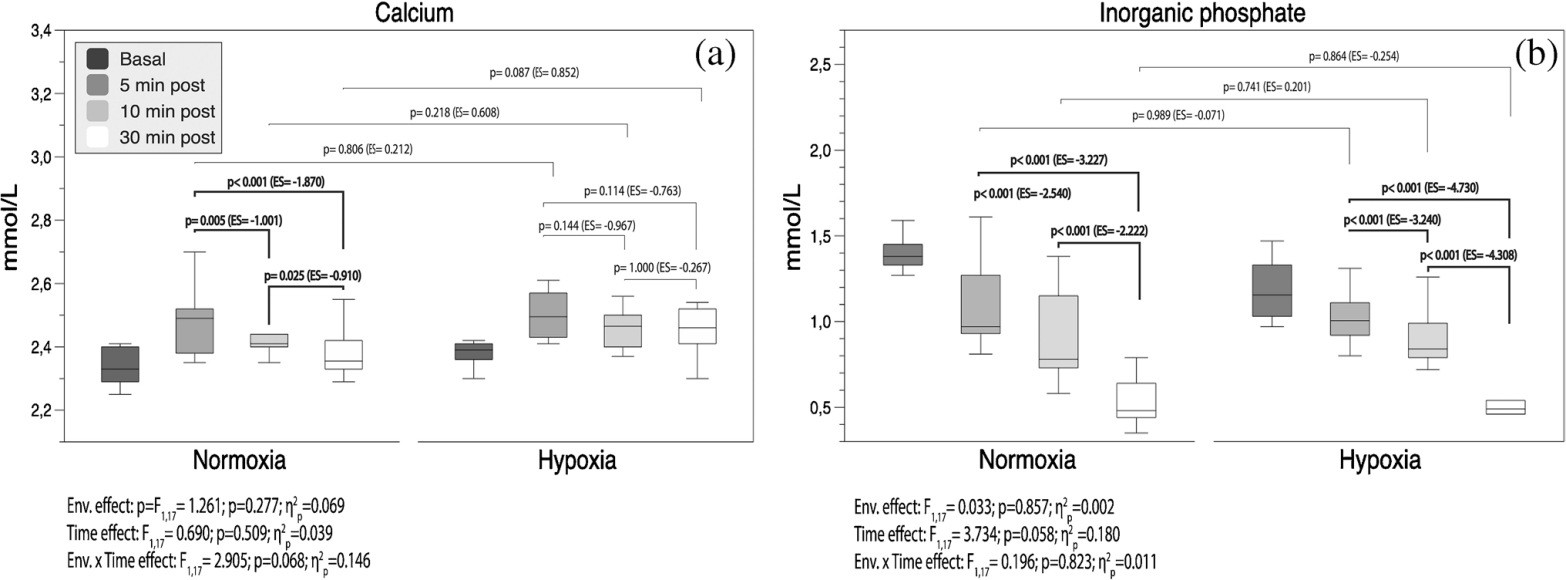
Circulating calcium (**a**) and inorganic phosphate (**b**) response throughout the 30 min of recovery. Boxes represent the median and interquartile range, whiskers minimum to maximum values. *p*-value (*p* < 0.05), partial eta squared from ANOVA (*η*^2^_*p*_), Cohen’s *d* effect size (ES). ES was calculated as (30–10 or 10–5 or 30–5 min post-exercise) or (HH-N) divided by the pooled standard deviation. Pre-exercise values were included as covariates

Figure 5a, b shows the serum Pi and Ca^2+^ levels throughout the 30-min recovery period. Post-exercise Ca^2+^ and Pi exhibited no differences between conditions (*p* > 0.05). Both groups showed large Pi removal from the bloodstream immediately after the R_T_ session (ES <  − 2.22; all *p* < 0.001).

## Discussion

This study aimed to analyse the impact of R_T_ in acute moderate HH on the muscle health. For this endeavour, the effect of a R_T_ session on TAC, myostatin, several metabolites and pro- and anti-inflammatory modulation was measured throughout 30 min of recovery in N and acute moderate HH conditions. The main findings revealed an immediate and proportional moderate to very large increase in circulating IL-10 and TNF-α after R_T_ in HH compared with N, suggesting a balanced pro/anti-inflammatory response with no clear impact on the metabolic, TAC and muscle atrophy biomarkers.

Combining the R_T_ session with the ascent in altitude is expected to intensify the metabolic stress response. Conversely, our results failed to find clear increases in circulating Pi and Ca^2+^ after exercise in HH. Compared with pre-exercise, there was a similar reduction rate in serum Pi in N and HH throughout the first 5 min following the R_T_ session. Consistently, other studies have reported no changes in circulating Pi concentrations 30 min after similar R_T_ exercise between normoxia and moderate hypobaric hypoxia [11]. Pi clearance happens in the first minutes of recovery and is associated with the normal restoration of cellular functional demands involved in the adenosine triphosphate (ATP) synthetic rate. The 5-min time window until the first blood draw in this study may not detect the expected changes in this analyte. Similarly to other research [11], our results revealed an immediate and large increase of serum Ca^2+^ after the R_T_ session regardless of the environmental condition (see Table 2). Although we did not find differences in the adjusted peak post-exercise Ca^2+^ value between conditions, the Ca^2+^ clearance was significantly higher at the end of the recovery period in N (ES =  − 1.87; *p* < 0.001) (Fig. 5a). Maintenance of circulating Ca^2+^ levels above baseline in HH throughout the recovery with respect to the N group could suggest a net increase in the absolute magnitude of the metabolic stress response in HH.

The available research has reported a fast IL-6 increase after R_T_ in N followed by a gradual reduction to baseline concentrations within 4 h [5, 14]. Additionally, R_T_ in hypoxia promotes anaerobic metabolism [18] and the upregulation of the inflammatory adaptative response. However, consistent with the absence of clear differences in the impact of the R_T_ session analysed on the metabolic stress response between N and HH, the release of IL-6 in this study displayed a large variability that limits conclusive results (Table 2 and Fig. 2a). Conversely, serum TNF-α concentration showed a large increase in the magnitude of the inflammation after R_T_ in HH throughout the recovery period with respect to N (Fig. 2c). Contrary to similar studies [5], the circulating TNF-α absolute delta change remained notably higher under HH compared with N (Table 2). Immediately after the R_T_ session in HH, IL-10 increased at 5 min post-exercise, and there inducing a progressive reduction in TNF-α secretion into the bloodstream [33] (see Fig. 2b, c). Benavente et al. [4] also found a moderate to large but non-significant increase in the absolute peak values of IL-6, IL-10 and TNF-α in acute HH after similar R_T_ exercise. The use of a longer rest period between sets (2 min) and the ingestion of a high-carb protein bar before R_T_ in the Benavente et al. study could contribute to mitigate the acute post-exercise inflammatory response [26] and partially explain the differences between the studies. Unfortunately, the absence of data related to this topic makes it difficult to compare our results in a more consistent way. An inflammatory response based on TNF-α may negatively impact muscle remodelling and activation of growth pathways [36]. However, there were no significant differences in the post-exercise IL-10/TNF-α ratio [34] between the groups, indicating that each group had a similar anti-inflammatory status. The greatest impact of R_T_ in HH on the serum IL-10 concentration suggests a larger modulation of the anti-inflammatory mechanisms [16] that must be further investigated. Notwithstanding, our data indicate a positive balance of the pro/anti-inflammatory mechanisms involved in counteracting the adverse consequences induced by a high-intensity R_T_ under acute HH conditions.

Previous research described significant suppression of myostatin messenger ribonucleic acid (mRNA) during recovery in HH compared with NH and N in endurance athletes [32]. Contrary, in accordance with the results of our study, in R_T_ exercise under acute NH, Britto et al. [5] observed an increase in myostatin mRNA within 4 h of exercise, concluding that the RTH seems to potentiate myogenesis through inflammation rather than protein balance alteration. Conversely, our results depicted a moderately lower rise of circulating myostatin after exercise in HH than in N (Table 2 and Fig. 3). Additionally, only HH depicted a moderate non-significant decline (ES =  − 0.72; *p* = 0.21) in myostatin levels 30 min post-exercise (Fig. 3). The regulation of myostatin expression can be explained, almost in part, by the overexpression of IL-10 and follistatin, both myostatin inhibitors [10]. Previous studies have reported a relationship between an increase in follistatin in response to R_T_ exercise and hypoxia exposure, among other factors [41]. Consistently, we found a large increase in serum IL-10 in HH following the R_T_ session while it remained similar to the pre-exercise in the N group (Fig. 2b and Table 2). The increase in IL-10 negatively affects the inflammatory response and consequently could restrict the increase in myostatin [19], as shown in the HH group, and stimulate muscle remodelling. In the Britto et al. [5] study, the IL-10 did not display any influence of exercise or hypoxic exposure. Differences in the procedure, such as the type of hypoxia (HH vs. NH), and the fact that recovery was conducted under normoxia conditions in the Britto et al. research, undoubtedly affect this result. Additional research is needed to elucidate the underlying mechanisms of the muscle remodelling process and cell differentiation pathways under hypoxic conditions.

Finally, a slight increase in TAC was observed in the N group after the exercise (Table 2). However, this increase was deemed of limited significance, as all recorded values for both groups remained well within the established normal range throughout the study [1]. Furthermore, consistent with Park et al. (36), serum TAC levels remained unchanged throughout the recovery in both conditions (Fig. 4). The stability in TAC levels did not impact on the inflammatory response through RONS-mediated signalling pathways.

This research has some limitations that should be noted: (1) only a few studies address the combination of HH and R_T_ protocols on muscle health–related parameters, which limits the comparison of our results. (2) Future studies should incorporate a crossover design to control intra-group variability. (3) Sample size was relatively small, which could influence the width of probability distributions across the outcomes. However, it must be considered that this population is of interest and is understudied, probably due to the high economical cost associated with the analysis of the procedure and the high personal cost associated with the data acquisition through vein canalisation. These results could be used for larger confirmatory studies.

## Conclusion

In summary, the results suggest the increment of the inflammation response after a R_T_ exercise in acute HH compared with the same exercise in N. However, the absence of a clear impact of the hypoxia on the metabolic, TAC and muscle atrophy biomarkers only allow a partial acceptance of the hypothesis of this study. In addition, HH also promoted a proportional large increase in IL-10 suggesting an anti-inflammatory response that ensures pro/anti-inflammatory balance. The potential association between the IL-10, myostatin and TNF-α response could favour muscle remodelling activity after a high-intensity exercise in HH, although this postulation requires further research.

## Data Availability

The data that support the findings of this study are available from the corresponding author upon reasonable request.
